# Brain size regulations by *cbp* haploinsufficiency evaluated by *in-vivo* MRI based volumetry

**DOI:** 10.1038/srep16256

**Published:** 2015-11-06

**Authors:** Juan C. Ateca-Cabarga, Alejandro Cosa, Vicente Pallarés, José P. López-Atalaya, Ángel Barco, Santiago Canals, David Moratal

**Affiliations:** 1 Center for Biomaterials and Tissue Engineering, Universitat Politècnica de València, Valencia, Spain; 2 Instituto de Neurociencias, Consejo Superior de Investigaciones Científicas, Universidad Miguel Hernández, Sant Joan d’Alacant, Spain

## Abstract

The Rubinstein-Taybi Syndrome (RSTS) is a congenital disease that affects brain development causing severe cognitive deficits. In most cases the disease is associated with dominant mutations in the gene encoding the CREB binding protein (CBP). In this work, we present the first quantitative analysis of brain abnormalities in a mouse model of RSTS using magnetic resonance imaging (MRI) and two novel self-developed automated algorithms for image volumetric analysis. Our results quantitatively confirm key syndromic features observed in RSTS patients, such as reductions in brain size (−16.31%, *p* < 0.05), white matter volume (−16.00%, *p* < 0.05), and corpus callosum (−12.40%, *p* < 0.05). Furthermore, they provide new insight into the developmental origin of the disease. By comparing brain tissues in a region by region basis between c*bp*^+/−^ and c*bp*^+/+^ littermates, we found that *cbp* haploinsufficiency is specifically associated with significant reductions in prosencephalic tissue, such us in the olfactory bulb and neocortex, whereas regions evolved from the embryonic rhombencephalon were spared. Despite the large volume reductions, the proportion between gray-, white-matter and cerebrospinal fluid were conserved, suggesting a role of CBP in brain size regulation. The commonalities with holoprosencephaly and arhinencephaly conditions suggest the inclusion of RSTS in the family of neuronal migration disorders.

The Rubinstein-Taybi syndrome (RSTS, MIM 180849)^1^ is a congenital, autosomal dominant disorder with rare incidence (1:100,000—1:125,000 human newborns)^2^,^3^,^4^. The clinical picture of the syndrome was first described in 1957^5^ and includes mental impairment, growth delay, skeletal abnormalities, hypertelorism, and microcephaly^6^,^7^. The main cause of RSTS are heterozygous mutations of the *CREBBP* gene (chromosome 16p13.3)^8^, which occurs in 40–60% of the diagnoses^3^,^9^,^10^. An alternative cause is a mutated *EP300* gene (22q13.2), which happens in 3% of the RSTS diagnoses^11^. *CREBBP* and *EP300* encode two paralog transcriptional co-activators with intrinsic lysine acetyltransferase (KAT) activity known as CREB binding protein (CBP) and p300^7^, respectively. Both proteins are highly similar and thought to play a major role in transcriptional regulation. Research in mouse models of RSTS, such as the *Crebbp* haploinsufficient mouse (*cbp*^+/−^)^12^ has revealed that these animals present similar features to patients, specifically cognitive impairment, growth delay and anatomical abnormalities. Additionally, it has been recently shown that CBP also plays a critical role in the regulation of adult neurogenesis^13^ and the differentiation of the cortical neural progenitor cells^14^.

A considerable number of studies and case reports have included brain imaging exams in RSTS patients^15^,^16^,^17^,^18^,^19^,^20^,^21^,^22^,^23^,^24^,^25^,^26^,^27^,^28^,^29^,^30^,^31^,^32^,^33^,^34^,^35^,^36^,^37^,^38^. These studies have consistently shown that microcephalia is a main feature in RSTS, as confirmed by a meta-analysis of 732 patients^39^. Other abnormalities, such as white matter abnormalities^25^,^26^,^31^, corpus callosum dysgenesis^17^,^24^,^26^,^33^,^35^ and abnormalities^20^,^27^, and cortical dysmorphologies^31^,^35^ including pachygiria^21^ and malgyration^35^ have been also reported. All these features may be correlated with the characteristic cognitive impairment. Preliminary brain images studies in mouse models of RSTS have also revealed some gross abnormalities in brain size^13^,^40^.

Most of these studies, both in mice and humans, were qualitative, and a precise description of the impact of CBP deficiency in brain development and structure is still lacking. To address this situation, we performed a quantitative volumetric study of brain structure in the RSTS mouse model of *Crebbp* haploinsufficient. Whereas traditional volumetry, also known as stereology, calculates these volumes based on histologic sections of the *ex vivo* brain, neurocomputational volumetry techniques allow the automatic analysis of brain images acquired *in vivo* by Magnetic Resonance Imaging (MRI) or Computed Tomography (CT), avoiding morphometric distortions introduced by histological tissue preparation^41^ and therefore providing a more precise quantification of brain anatomy^42^. We have used two in-house developed algorithms for automated volumetry analysis based on MRI images acquired at 7T to unveil the selective impact of CBP deficiency in the development of different brain regions. We provide novel evidences suggesting that the brain of RSTS mice present holoprosencephaly, composed of important dysgenesis in the olfactory bulb and cortex and suggestive of an underlying neuronal migration disorder. The latter conclusion is also supported by case reports in RSTS patients reporting cortical abnormalities.

## Materials and Methods

### Animal preparation

C*bp*^+/−^ mice^12^ (CBP group) and control littermates (WT group) were generated as previously described^13^. Adult mice (95 to 118 days old, WT group: 104,17 ± 7,70 days old, CBP group: 106,00 ± 7,00 days old) were weighted 14.5 to 28 g (WT group: 24.6 ± 2.7 g, CBP group: 17.8 ± 2.3 g, total: 21.5 ± 4.3 g). These mice are maintained on a DBA and C57BL/6J mixed background because c*bp*^+/−^ mice are not viable in a pure C57BL/6J background^40^,^43^. Mice were maintained according to animal care standards established by the European Union and all the protocols were approved by the Institutional Animal Care and Use Committee and carried-out in accordance with the approved guidelines.

### Image acquisition

**Figure 1:**
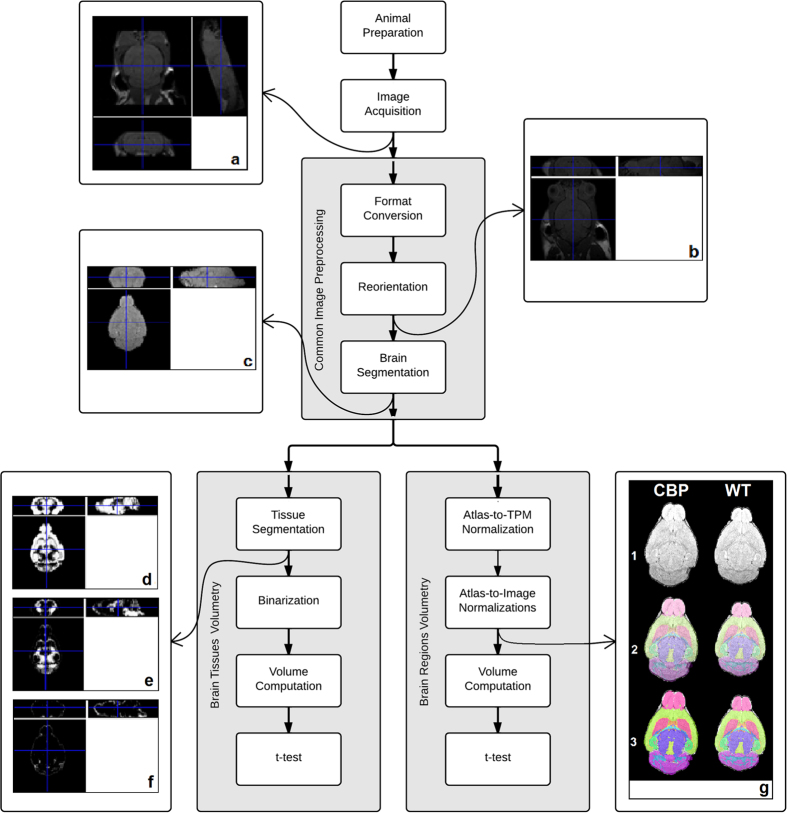
Block diagram and intermediate results of the methods. The preparation of the 11 animals (6 WT, 5 CBP) is followed by the acquisition of magnetic resonance images (Panel (**a**)). The common image preprocessing stage is composed by format conversion, reorientation (Panel (**b**)), and brain segmentation, which produces an extracted brain image (Panel (**c**)). At this point, the procedure splits into two image processing algorithms. Brain tissues volumetry further segments the previously extracted brain into three substances (grey matter, white matter, and cerebrospinal fluid, Panels (**d**–**f**)) and obtains their volumes for all the subjects. On the other hand, brain regions volumetry implies a combination of normalizations to fit the atlas to each of the brain images in order to get the regional volumes of the subjects (Panel (**g**)). Progressive overlappings (1–3) of segmented regions over brain axial slices from selected CBP (Panel (**g**), left column) and WT (Panel (**g**), right column) subjects are shown. All the resulting volumes were compared by means of t-tests.

Before the image acquisition, the animals were anesthetized with isoflurane. During the acquisition, the head of the animal was situated in a stereotactic frame while temperature, cardiac frequency and oxygen saturation (SpO_2_) were monitored. The images were acquired with a 7-T MRI scanner (Bruker BioSpin, Ettlingen, Germany) under coronal 3D T1-weighted configuration, using an EPI sequence with the following parameters: TR = 360 ms, TE = 10 ms, 35 averages, in plane resolution 86 × 86 μm, slice thickness 0.35 mm, with a matrix size of 256 × 256, obtaining 17 DICOM images (Fig. 1a).

### Common image preprocessing

The common image preprocessing stage consisted in three tasks. Firstly, the DICOM images were converted to the NIfTI format. Afterwards, in order to accommodate them to the software that implements the two image processing algorithms (further detailed below), the images were reoriented by means of the FMRIB Software Library (FSL)^44^ (Oxford Centre for Functional MRI of the Brain, Oxford, United Kingdom) (Fig. 1b). Finally, brain segmentation was performed by means of the Brain Extraction Tool (BET)^45^, which is also included in FSL. This operation basically consists in skull-stripping by fitting a deformable model to the brain surface, thus producing the clean intracranial volume used for brain tissues and brain regions volumetry (Fig. 1c).

### Brain Tissues Volumetry

For the volumetric analysis of the brain tissues, the already extracted brain images were segmented in their three constituent tissues, i.e., grey matter (GM), white matter (WM) and cerebrospinal fluid (CSF) (Fig. 1d–f). This process was carried out with the Unified Segmentation algorithm^46^, included in the Statistical Parametric Mapping (SPM) software (Wellcome Trust Centre for Neuroimaging, London, United Kingdom). This algorithm also spatially normalizes the images with the assistance of mouse brain tissue probability maps (TPM)^47^. Each one of the three resulting images is composed by voxels whose intensity indicates the probability of belonging to the correspondent tissue. In order to render the voxels that most probably belong to each of the three tissues, the three segments were binarized. Lastly, a self-developed algorithm was run to compute the volume of each tissue by summing all the belonging voxels and multiplying by the voxel volume. Two-tailed Student’s t-tests were applied in order to show significant differences in the volume values between CBP and WT groups.

### Brain Regions Volumetry

For the brain regions volumetric analysis, the already extracted brain images were segmented in their 43 constituent regions, according to the Kovačević atlas of the mouse brain^48^. This process was carried out by superposing the atlas to each individual brain image, which was done by a combination of spatial transformations. Firstly, the atlas was spatially normalized from its native space to the space of the TPM and then the atlas in the space of the TPM was spatially normalized in order to adapt to each of the 11 image spaces, each one from one individual (Fig. 1g). Finally, in a similar way to the brain tissue volumetry, the volume of each region was computed by summing all the belonging voxels and multiplying by the voxel volume prior to two-tailed Student’s t-tests that show significant differences among both groups.

## Results

While there have been few *in vivo* volumetry studies in mouse models of intellectual disability disorders^42^,^49^, to the best of our knowledge we have developed and applied the first automated volumetry analysis of the mouse brain conducted *in vivo*.

### Brain tissue volumetry

**Figure 2:**
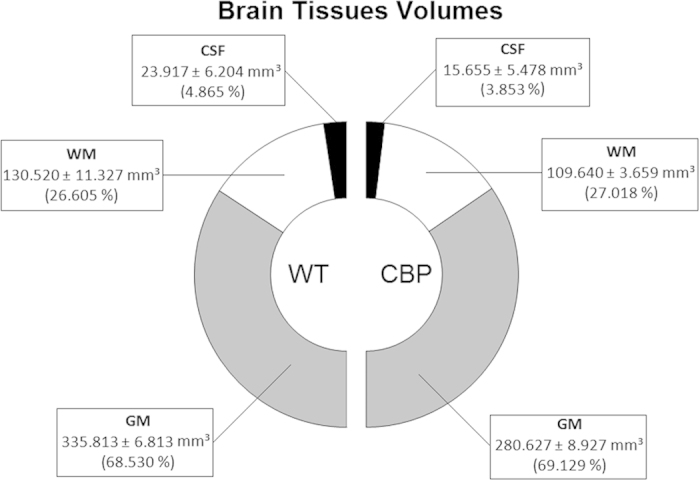
Results of the brain tissues volumetry analysis. Volumes (mean ± standard deviation) of the three brain tissues (Grey Matter, White Matter, and Cerebrospinal Fluid) and their ratios are represented for Wildtype (6 subjects) and CBP group (5 subjects). Both groups show similar proportions of the three matters, which allows us to conclude that Rubinstein-Taybi Syndrome has no effect in terms of brain tissues proportion.

We report the volumes of gray matter (GM), white matter (WM), and cerebrospinal fluid (CSF) from 11 subjects (5 c*bp*^+/−^ mice and 6 wild type littermates). Results reveal that the brains of CBP group are smaller than those of the WT group (Fig. 2). Specifically, GM, WM, and CSF volumes are significantly lower in CBP subjects (−16.43% [*p* < 0.0001], −16.00% [*p* = 0.005], and −34.55% [*p* = 0.044], respectively). Similarly, brain volume, composed by GM and WM, significantly changed by −16.31% (p < 0.0001) whereas total intracranial volume (TICV), formed by the brain and CSF volumes, significantly changed by −17.20% (*p* < 0.0001). In contrast to the absolute volume values, the proportions between the three tissue classes are kept constant in both groups. Regarding brain tissue ratios, the GM- and WM-to-brain-volume ratios along with GM-, WM-, CSF- and brain-to-TICV ratios did not significantly change between groups (*p* > 0.05). In other words, the brains are reduced in volume but the ratios are unaltered, which leads us to preliminary conclude that, CBP hemydeficiency provokes a down-scaling effect in the brain.

### Brain regions volumetry

We report the volumes of 43 brain regions (according to the Kovačević atlas of the mouse brain^48^) for each of the 11 subjects. In 41 of the 43 regions of the CBP group, a volume reduction was observed (obtaining a volume change of −10.5 ± 4.6% [mean ± standard deviation]), being significant (*p* < 0.05) in 25 of the 41 regions (mean volume change of −13.34 ± 1.41%).

**Figure 3:**
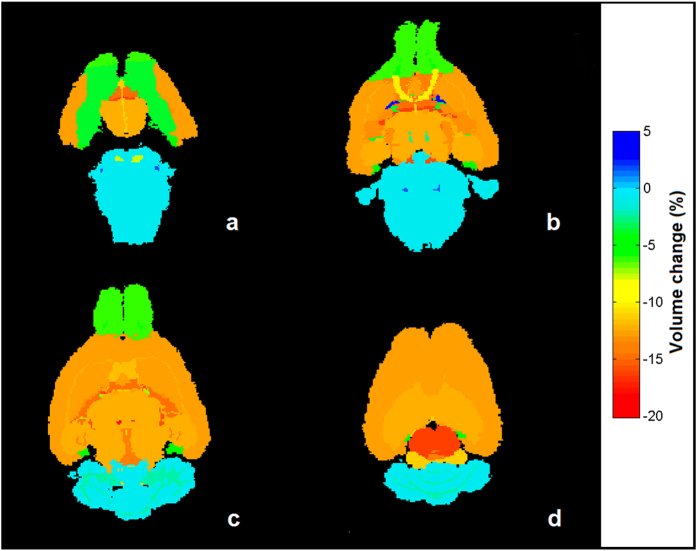
Results of the brain regions volumetry analysis. Colored maps indicating regional volume change (in %) of CBP group mean brain with respect to WT group are shown at axial slices *Z* = 2 (a), *Z* = 5 (b), Z = 9 (c), and *Z* = 14 (d) of the three-dimensional magnetic-resonance image. Brain regions can be grouped in three zones according to their behavior: regions evolved from the embryonic rhombencephalon that experiment no significant volume change (−0.68 ± 0.21%, blue regions), olfactory bulb that shows a low level of volume reduction (−6.28%, green regions), and regions developed from prosencephalon, which show a higher reduction (−12.51 ± 0.36%, orange regions).

When the changes in volume per region were mapped on the mouse brain atlas (Fig. 3), we identified three zones of different developmental origin showing differential behaviors. First, the posterior part of the brain (cerebellum and brain stem), which is derived from the embryonic rhombencephalon, showed no significant reduction in volume (−0.68 ± 0.21%). Second, regions that are developmentally derived from the embryonic prosencephalon, such as the telencephalon and diencephalon, showed the largest reduction in volume (−12.51 ± 0.36%). Finally, the anterior part of the brain (olfactory bulb) showed a lower but significant volume reduction (−6.28%). Interestingly, our quantification reveals that cortical and corpus callosum volumes are equally reduced (−12.79% and −12.40% respectively).

### Validation of volumetric results

In order to validate the results from the volumetric analysis of the brain tissues and regions using the novel algorithms, we compared our results with those from Kovačević *et al.*^48^. The volumes reported for the WT group are coincidental with those presented by them. The small differences in absolute volume values may result from the fact that Kovačević and colleagues used inbred mice whereas we used a mixed C57/DBA background. Besides, Kovačević *et al.* analyzed images from *ex vivo* mouse brains while this work has dealt with *in vivo* brain images.

## Discussion

Our results show two novel observations. First, consistent with studies in humans, we quantitatively confirm the existence of microcephaly and WM abnormalities in the brain of *cbp+/−* mice. We quantitatively confirm a drastic reduction (−16.31%) in brain volume associated to *cbp* haploinsufficiency, a finding in good agreement with the characteristic microcephaly associated to RSTS^39^. Our results further show that microcephaly is not only produced by GM hypoplasia, since a concomitant and proportional decrease (−16.00%) of WM volume was also found. Previous studies and case reports support this conclusion showing WM abnormalities^25^ and volume reductions^26^,^31^ in RSTS patients.

Second, our results suggest for the first time that CBP plays a differential role in the control of the development of prosencephalic regions, including the olfactory bulb and neocortex. Interestingly, in these areas tissue reductions kept a constant proportion, which suggests a role for CBP in brain size regulation. These results quantitatively confirm key syndromic features of RSTS, such as microcephaly, as well as anomalies in the WM, corpus callosum, and cortex. In particular, our results show that the regions evolved from the embryonic prosencephalon are greatly reduced in *cbp*^+/−^ subjects (−12.51 ± 0.36%). This could point to a mild form of holoprosencephaly (HPE, MIM 236100), which has been already linked with RSTS^57^,^50^. HPE is also associated with Dandy-Walker malformation (OMIM 220219)^51^, that has been also reported in RSTS patients^29^,^34^,^35^,^37^,^52^,^53^, and facial dysmorphism^50^, found in this RSTS mouse model^13^. The volumetric results in the prosencephalon drive two important observations. On the one hand, the volume of the cortex is greatly reduced in *cbp*^+/−^ animals, pointing to the diagnosis of cortical atrophy. On the other hand, the olfactory bulb is also reduced (although less dramatically), what could be related to arhinencephaly, a diagnose that describes hypoplasia or absent olfactory tracts and bulb and that has been equally linked to HPE and RSTS^57^,^50^. Interestingly, HPE and arhinencephaly share an underlying etiology: they are both considered neuronal migration disorders (NMD). A perturbed neuronal migration mechanism could be the product of a failure in the neurogenesis or lack of differentiation of the progenitor cells to produce the three neural lineages (neurons, astrocytes, and oligodendrocytes). In fact, such defects have been recently shown to occur in *cbp* haploinsufficiency during development^14^,^54^. Furthermore, CBP deficiency is also associated with a reduction of the number of newborn neurons in response to environmental enrichment in adult mice^13^.

Neuronal migration is a fundamental process in the development of the cortex, which in normal conditions is composed by six layers, created in an inside-out manner by means of subsequent waves of radially migrating neurons. NMDs are known to cause an abnormally thin cortex^55^, and cortical defects like (micro)lissencephaly, (poli)microgyria and pachigyria, observed in RSTS^21^. Furthemore, malgyration and microscopic abnormalities of the cytoarchytecture of the cerebral cortex^35^ along with “abnormal cortical infoldings”^31^ have also been reported in RSTS patients. NMD-caused microlissencephaly is also shown to contribute to microcephaly^55^, a feature often found in RSTS patients and reported here for the first time in *cbp*^+/−^ mice.

More research in RSTS needs to be done to clarify the causes of this congenital disorder. Effective clinical diagnosis is fundamental for the early detection of RSTS. While there exist RSTS clinical guidelines^3^,^4^, there is a lack of established diagnostic criteria for the disease^21^ and the inclusion of neuroimaging studies in the clinical workflow has been proposed^15^,^17^,^18^,^21^,^22^,^23^,^25^,^29^,^37^,^56^. Our results provide strong evidence in support of neuroimaging-assisted diagnosis to reach early detection and treatment of RSTS-associated disorders. Furthermore, they offer neurobiological insight into the role of CBP in normal brain development, and the consequences of its haploinsufficiency in RSTS models. The advance in the field will contribute to a better understanding of the disease and will help its diagnosis.

## Additional Information

**How to cite this article**: Ateca-Cabarga, J. C. *et al.* Brain size regulations by *cbp* haploinsufficiency evaluated by *in-vivo* MRI based volumetry. *Sci. Rep.* **5**, 16256; doi: 10.1038/srep16256 (2015).
